# Exosomes in the lung cancer microenvironment: biological functions and potential use as clinical biomarkers

**DOI:** 10.1186/s12935-021-01990-0

**Published:** 2021-06-30

**Authors:** Runzhi Qi, Yuwei Zhao, Qiujun Guo, Xue Mi, Mengqi Cheng, Wei Hou, Honggang Zheng, Baojin Hua

**Affiliations:** 1 Department of Oncology, Guang’anmen Hospital, China Academy of Chinese Medicine Sciences, Xicheng District, Beijing, China; 2 Beijing University of Chinese Medicine, Chaoyang District, Beijing, China; 3 Shaanxi University of Chinese Medicine, Qindu District, Xianyang, Shaanxi China

**Keywords:** Exosome, Lung cancer, Tumour microenvironment

## Abstract

Lung cancer is one of the most common malignant tumours worldwide. however, emerging immunotherapy and targeted therapies continue to show limited efficacy. In the search for new targets for lung cancer treatment, exosomes have become a major focus of research. Exosomes play an important role in the tumour microenvironment (TME) of lung cancer and affect invasion, metastasis, and treatment responses. This review describes our current understanding of the release of exosomes derived from different cells in the TME, the effects of exosomes on T/Tregs, myeloid-derived suppressor cells, tumour-associated macrophages, dendritic cells, and natural killer cells, and the role of exosomes in the endothelial–mesenchymal transition, angiogenesis, and cancer-associated fibroblasts. In particular, this review focuses on the potential clinical applications of exosomes in the lung cancer microenvironment and their prognostic and diagnostic value.

## Background

Lung cancer is one of the most commonly diagnosed cancer and the leading cause of cancer deaths in both sexes combined [1], despite improvements in diagnosis and treatment, such as the emergence of immune checkpoint inhibitors, new-generation drugs (e.g. EGFR-TKI, ALK, eml4), and advanced radiotherapy technology. The recurrence of non-small cell lung cancer (NSCLC) remains high, with 5-year overall survival rates ranging from 83% for stage IA to 36% for stage IIIA [2], and the 5-year survival rate for patients with small cell lung cancer (SCLC) remains fairly low at only 10% [3].

To find effective treatments and overcome low immunotherapy efficiency and drug resistance, increasing research has focused on the lung cancer microenvironment. The vasculature, immune and inflammatory cells, extracellular matrix (ECM), and cancer-associated fibroblasts (CAFs) are major components of the tumour microenvironment (TME), which is recognised as a target-rich landscape for the development of novel agents in lung cancer [4, 5]. Interactions between these components and cancer cells contribute to angiogenesis, intravasation, and metastasis in lung cancer. The key molecules by which cancer cells cooperate with the microenvironment are candidate biomarkers or drug targets [6].

Among these functional mechanisms, exosomes carrying large amounts of information and molecules play an important role in intercellular communication and are indispensable mediators of various processes in the TME [7]. Exosomes were named in 1987 and were first described by Johnstone et al. in 1983 as small vesicles released from maturing sheep reticulocytes containing externalised protein components of reticulocytes. They were later recognised as important regulators of cell function and intercellular communication.

Exosomes transport proteins (cytosolic and transmembrane proteins), lipids, and nucleic acids (microRNAs [miRNAs], mRNAs, long non-coding mRNAs, and DNA) to target cells, thus regulating their behaviour [8–10]. Exosomes, with a diameter of 30–100 nm, are intraluminal vesicles formed by the inward budding of the endosomal membrane and are secreted after fusion on the cell surface [11]. In this review, we have described the effects of exosomes on various cell types in the lung cancer microenvironment, with an emphasis on the implications for diagnosis and treatment.

## Origin and characteristics of exosomes

Exosomes form by inward budding in the plasma membrane, and are classified as early and late endosomes, known as multivesicular bodies. Numerous intraluminal vesicles form by the invagination of multivesicular body membranes. Exosome release is regulated by a number of genes and proteins. For example, miR-134 and miR-135b precisely regulate YKT6 expression in lung cancer cells, which in turn controls exosome release [12]. Furthermore, exosomes are produced by a variety of cells in the lung TME, including lung cancer cells, cancer-associated fibroblasts (CAFs), tumour-associated macrophages (TAMs), and dendritic cells (DCs). CAFs play a critical role in the epithelial–mesenchymal transition (EMT), which is associated with malignant progression, via exosomes, and exosomal SNAI1 is crucial for this process. Exosomes derived from macrophages also regulate lung cancer cell proliferation in the TME [13]. Duan et al. found that let-7a-5p could be transported from macrophages to lung cancer cells as a macrophage exosome cargo and could directly target BCL2L1, thereby promoting A549 cell autophagy and cell death via the PI3Kγ pathway. Expression levels of the lung cancer marker proteins MYC, EGFR, and vimentin are also altered by the aberrant expression of BCL2L1 [14]. The miRNAs miR-193a, miR-210, and miR-5100 are transferred by exosomes derived from bone marrow-derived mesenchymal stem cells (BMSCs) to neighbouring cancer cells, thereby activating STAT3 signalling and promoting cancer cell invasion and EMT [15]. Xu et al. found that microvascular endothelial cell exosomes promoted tumour cell survival via the upregulation of S100A16 in the microenvironment of SCLC with brain metastases, which are associated with a poor prognosis [16]. DCs play a critical role in adaptive immunity in lung cancer. They are indispensable for the response to checkpoint inhibitor immunotherapy [17]. There are at least two phenotypes of DC-derived exosomes: bone marrow mature DC-derived exosomes and bone marrow immature DC-derived exosomes. Researchers have identified 139 miRNAs in mature DC-derived exosomes with key roles in cell biology and function [18, 19]. T lymphocyte- and B lymphocyte-derived exosomes also play important roles in the immune microenvironment. Recently, B lymphocytes have been shown to facilitate the impaired function of CTL by enzymatic activities in B lymphocyte-derived exosomes [20]. Additionally, chimeric antigen receptor (CAR)-T cells release exosomes that carry CAR on their surface and inhibit tumour growth [21]. Exosomes can be produced by a variety of immune cells in the TME; however, the mechanism of action of these exosomes in lung cancer requires further study.

## Exosome targeting and uptake

Exosomes can be ingested by a variety of cells in the TME and exert functions via proteins, nucleic acids, and other substances. First, the recognition of exosomes requires membrane protein interactions. CD169 recognises exosomes and mediates the immune response to exosome antigens. The adhesion of B cell-derived and DC-derived exosomes is CD169-dependent [22]. After recognising exosomes, some proteasome components, such as Tim family members Tim1 and Tim4, bind by exosomal phosphatidylserine [23]. Additionally, exosomes express chemokines, including CCLs and CXCLs, to attract leukocytes, providing an alternative explanation for exosome cellular recognition [24]. After recognition, exosomes are internalized into recipient cells. Tetraspanins on exosomes are part of the antigen recognition system and contribute to the activation of signalling pathways induced by exosomes. The merging of the exosome cytosol and cytoplasm is achieved by membrane fusion at the plasma membrane or by uptake followed by fusion with the endosomal membrane [18]. Exosomes are taken up by many endocytic pathways, including clathrin-mediated endocytosis, caveolin-dependent endocytosis, macropinocytosis, phagocytosis, and lipid rafts [25, 26]. Caveolin-1 and clathrin heavy chain mediate caveolae-dependent endocytosis and clathrin-dependent endocytosis, respectively. The absorption of exosomes is reportedly dependent on the recipient cell and not on exosomal surface molecules, and this process can be visually observed by immunofluorescence microscopy[27, 28]. Extensive studies of exosome targeting and uptake have provided a basis for the development of new clinical treatment methods and dosage forms.

## Functions of exosomes in the lung cancer microenvironment

### Tumour-associated macrophages regulation by lung cancer-associated exosomes

Tumour-associated macrophages (TAMs) have been linked to lung cancer cell initiation, progression, and metastasis in the TME [29]. The key mechanisms underlying such functions are classical activation and alternative activation of macrophages. The classical activation (M1) of macrophages is characterised by the production of antineoplastic and proinflammatory macrophages, whereas alternative activation (M2) is related to immunosuppression, tumourigenesis, and angiogenesis [30]. Exosomes promote macrophage polarisation, resulting in immunosuppression, angiogenesis, and tumour progression. NSCLC exosomes can induce M0 macrophages and myeloid-derived suppressor cells to differentiate into M2 macrophages. Additionally, one study also confirmed that exosome induced M2 polarization might not be p53 gene dependent [31]. In a recent study, hypoxia stimulated tumour-derived exosome secretion promoted oxidative phosphorylation in TAMs by transferring let-7a miRNA and by suppressing the insulin-Akt-mTOR pathway. Hypoxia-induced exosomes enhance macrophage recruitment and promote M2-like polarisation in vitro and in vivo [32]. Another study showed that exosomes from hypoxic lung cancer cells polarized macrophages to M2-type via miR-103a. Exosomal miR-103a decreased PTEN levels or increased Akt/Stat3 activation and several immunosuppressive factors [33]. By studying SK-LU-1 lung adenocarcinoma cell derived exosomes and J774 macrophages, Trivedi's team found that the expression of exosomal miR-125b et al. from SK-LU-1 cell were increased by double-targeted (wild-type p53 and microRNA-125b) transfection of hyaluronic acid-based nanoparticles. At the same time, J774 macrophages treated with these exosomes repolarized towards M1 phenotype (pro-inflammatory and antitumour type) [34]. Tumour-derived exosomes influence the phenotype and function of macrophages. Many studies have focused on the effect of tumour-derived exosomes on TAMs, while the interaction between macrophages and other immune cells via exosomes remains to be further studied.

## Dendritic cells regulation by exosomes in the TME of lung cancer

Dendritic cells (DCs) play an essential role in the regulation of tumour-specific immune responses. DC-based immunotherapy is unsatisfactory due to the poor immunogenicity of cancer cells and low uptake efficiency of antigens, even though DCs are the most potent antigen-presenting cells [35]. Lung tumour cell-associated exosomes can more effectively deliver a variety of tumour antigens to DCs [36]. Wang et al. have reported that tumour-associated exosomes stimulate DC maturation and enhance MHC cross-presentation, which directly promotes a tumour-specific cytotoxic T lymphocyte response. Exosomes also reduce PD-L1 expression on DCs, resulting in a decrease in the Treg population [37]. Additionally, in vitro analyses have shown that DC-derived exosomes can transfer MHC class I and II complexes to DCs and trigger CD8+ and CD4+ T lymphocyte activation [38, 39]. It has been shown that exosomes activate DC maturation, while DCs incubated with exosomes derived from Rab27a-overexpressing cells promote CD4+ T cell proliferation [40].

## Interaction between NK cells and exosomes in the TME

Natural killer (NK) cells are independent, non-specific immune cells. They can directly kill tumour cells without MHC restriction to the target [41]. However, tumour cells impact normal functions of NK cells and impair cytotoxicity in the TME. The degree of NK cell infiltration is positively related to the survival rate in lung cancer [42]. Decreased expression of the NK cell-activated receptor NKG2D in the TME indicates immune tolerance [43]. Hypoxia inhibits the immune function of NK cells. Exosomes derived from hypoxic tumour cells transfer TGF-β1 to NK cells and inhibit NK cell function by suppressing NKG2D. Additionally, miR-23a in hypoxic exosomes acts as an immunosuppressive factor by inhibiting CD107a expression in NK cells [44]. NKG2D endocytosis decreases the expression of surface receptors and regulates signalling in NK cells [45]. Exosome-associated NKG2D ligand could combine with NKG2D, triggering signalling in NK cells [46]. DNAX accessory molecule-1 (DNAM1), which functions like NKG2D, is another key receptor of NK cells. DNAM1 is expressed more in infiltrating NK cells of primary lung tumours compared to the expression in surrounding normal tissues. NK cells have a cytolytic effect in lung tumours via exosomal DNAM1 receptor-ligand binding and endocytosis [47].

## Treg/T cell regulation by exosomes in the TME

The proportion of CD4^+^CD25^+^Foxp3^+^ T regulatory cells (Tregs) and functional alterations of T lymphocyte subsets in the TME are critical for the immune escape of lung cancer cells [48]. Lung cancer cell-derived exosomes act on DCs, thereby increasing Treg differentiation in the TME, decreasing the proportion of CD4+ T cells, and decreasing IFN-γ production. However, PD-L1 blockage partially modulates the exosome-induced DC-associated immunosuppressed microenvironment [49]. PD-L1-mediated immunotherapy is widely used in clinical settings. Exosomal PD-L1 is a new target for the regulation of T cell immune function and the tumour immune microenvironment. Tumour cell-derived exosomal PD-L1 suppresses T cell activation and contributes to immunosuppression. However, blocking of exosomal PD-L1 and anti-PD-L1 antibodies can inhibit tumour growth [50, 51]. In addition, Microsatellite instability (MSI)is also closely associated with PD-1/PD-L1 expression and MSI tumours have high immunogenicity [52]. The transfer of exosomes containing Let-7d from Tregs to helper T lymphocyte 1 (Th1) cells contributes to the prevention of diseases, thereby providing a mechanism underlying Treg-mediated immunosuppression by miRNA-containing exosomes [53]. Epidermal growth factor receptor (EGFR) is closely related to lung cancer. Huang et al. found that exosomes containing EGFR could induce tolerogenic DCs and generate tumour antigen-specific Tregs [54]. The oncogenic Ras protein Kirsten rat sarcoma viral oncogene homolog (KRAS) is frequently mutated in lung cancer. CD4^+^ naïve T lymphocytes incubated with tumour-derived exosomes from mutant KRAS^+/+^ NSCLC cells induce Foxp3^+^ Treg generation by phenotypic switching. Foxp3 regulates Treg functions [55]. This conversion is related to IFN signalling, which eventually results in immunosuppression [56].

## Myeloid-derived suppressor cells interact with exosomes in the TME

Myeloid-derived suppressor cells (MDSCs) are divided into the following two groups: M-MDSCs, which are morphologically similar to monocytes, and PMN-MDSCs, which resemble polymorphonuclear cells [57]. MDSCs require the activation of inflammatory cytokines, such as IL-6 and TNF-α, and tumour-associated cytokines, such as GM-CSF and M-CSF, to form a population of immunosuppressive cells [58]. MDSCs regulate T cell function to form an immunosuppressive microenvironment, and exosomes with cytokines play a role in this process. Tumour exosomal PGE2 and TGF-β strengthen the induction of MDSCs, activate the upregulation of Cox2, IL-6, VEGF, and ARG-1 in MDSCs, and promote T-exosome-mediated tumour proliferation [59]. MDSCs are the main target cell population of exosomes from lung cancer cells. MDSCs internalise lung cancer-derived exosomes together with soluble factors (miR-126-3p, miR-27b, miR-320, and miR-342-3p) and upregulate the expression of suppressive molecules, including ARG-1 and TGF-β [60]. Little is known about the role of exosomes in MDSCs, and there is no direct evidence that exosomes from lung cancer cells can directly regulate the phenotypes and functions of MDSCs.

## Regulation of epithelial-mesenchymal transition by lung cancer-derived exosomes

Epithelial-mesenchymal transition (EMT) is a key mechanism for initiating lung cancer cell invasiveness and metastasis [61]. Exosomes relay signals from CAFs to lung cancer cells and play an important role in EMT [62]. A recent study has revealed that CAFs deliver SNAI1 exosomes to lung cancer cells to induce EMT. Markers of EMT, including E-cadherin, vimentin, and α-smooth muscle actin (α-SMA), are upregulated during exosome-induced EMT [13]. *ZEB1* mRNA is a major EMT transcription factor in mesenchymal cells in NSCLC. Oncogenic exosomes derived from mesenchymal NSCLC cells can transfer chemoresistance and mesenchymal phenotypes to recipient cells by *ZEB1* mRNA in exosomes [63]. These results provide the mechanism by which parental epithelial cells transform into mesenchymal lung cancer cells with the chemoresistance phenotype. miRNAs in exosomes also affect lung cancer carcinogenesis and metastasis. One of these miRNAs, miR-499a-5p, is upregulated in lung cancer cell lines and their exosomes. Tumour-derived exosomal miR-499a-5p has diagnostic and therapeutic value and promotes EMT via the mTOR signalling pathway in lung cancer [64]. Exosomal miR-9 from lung cancer cells effectively acts on HUVECs by downregulating the SOCS5-JAK-STAT pathway, which promotes endothelial cell migration and angiogenesis [65]. Increasing research has demonstrated the important role of bone marrow-derived mesenchymal stem cells (BMSCs) in EMT. Exosomes derived from BMSCs, which are components of the lung cancer microenvironment, mediate the transfer of miR-193a-3p, miR-210-3p, and miR-5100 and promote cancer cell invasion and EMT by activating STAT3 signalling [15]. These results suggest that transcription factors, mRNAs, and microRNAs in exosomes all play important role as mediators of EMT, thereby promoting lung cancer cell invasion, infiltration, and metastasis.

## Cancer-associated fibroblasts regulation by lung cancer-derived exosomes

Cancer-associated fibroblasts (CAFs) are a major cellular component of TME in most solid cancers. Lung cancer cells can transform the phenotype of fibroblasts via exosomes and related factors. Exosome-associated miR-142-3p promotes the transformation of lung fibroblast cells to CAFs via TGF-β signalling [66]. Interacted, CAFs also promote tumour cells proliferation. It is generally believed that CAFs promote tumour angiogenesis. Some studies have shown that exosomes from patients with lung cancer can induce cancer cell reprogramming [67]. Exosomes that overexpress miR-210 can activate the functions of CAFs and increase the expression of proangiogenic factors, such as MMPs, FGF2, and VEGFA [68]. CAFs deliver the transcription factor SNAI1 to lung cancer cells via exosomes, thereby inducing epithelial transformation via *CDH1* encoding E-cadherin and *VIM* encoding Vimentin [13]. In terms of energy metabolism, exosomes supply nutrients to starving cancer cells by a mechanism similar to micropinocytosis. CAF-derived exosomes with amino acids, lipids, and TCA-cycle intermediates [69, 70] are ingested by cancer cells for central carbon metabolism and promote tumour growth under nutrient deprivation or nutrient stress conditions [71]. In addition to controlling somatic cell senescence, telomerase can also inhibit lung cancer cells. Telomerase is activated in more than 90% of the cancer cells [72, 73]. Similarly, *hTERT* mRNA has been detected in exosomes isolated from sera of patients with lung cancer. The transfer of exosomal telomerase from cancer cells into fibroblasts may contribute to alterations in the TME [74].

## Angiogenesis regulation by exosomes in the TME

Various studies have evaluated the role of hypoxia during interactions between immune cells and EMT components. Lung cancer-derived exosomes increase under hypoxic conditions and play a critical role in angiogenesis. miRNAs in lung cancer cell-derived exosomes have important functions under hypoxic conditions [75]. Exosomal miR-23a targets prolyl hydroxylase 1 and 2 (PHD1 and 2) and suppresses expression in endothelial cells. Hypoxia-inducible factor-1α (HIF-1α) accumulates in endothelial cells, thereby increasing angiogenesis. Additionally, exosomal miR-23a inhibits the tight junction protein ZO-1, which is related to vascular permeability and cancer cell migration [76]. Radiotherapy, one of the most important treatment approaches in lung cancer, also affects exosome production. In an in vitro experiment, exosomes released from γ-irradiated cells or hypoxic cells activated lung tumour progression. Furthermore, angiopoietin-like 4 (ANGPTL4) derived from exosomes contributes to angiogenesis, suggesting that it is a potential diagnostic biomarker of lung cancer [77]. The process of tumour angiogenesis is also closely related to fibroblasts and their proangiogenic factors. According to Fan et al., miR-210 is encapsulated in exosomes, secreted by lung cancer cells, and eventually acts on fibroblasts. The ten-eleven translocation 2 (TET2) and JAK2/STAT3 signalling pathway of CAFs is a target of miR-210 in the process of angiogenesis, which promotes the release of the proangiogenic factors vascular endothelial growth factor (VEGF), MMP9, and FGF2 [68]. The overexpression of tissue inhibitor of metalloproteinase-1 (TIMP-1) also leads to the accumulation of miR-210 in exosomes and thus promotes angiogenesis [78]. The STAT3 signalling pathway is not only a target for exosomal miRNAs in the promotion of angiogenesis but also boosts the release of miRNAs. STAT3 upregulates exosomal miR-21 levels in transformed HBE cells. Interestingly, miR-21 in exosomes further activates the STAT3 signalling pathway in HBE cells, which increases VEGF levels and induces tumour angiogenesis [79]. Based on the important effect of exosomal miRNAs on angiogenesis, they have been evaluated as treatment targets in NSCLC. For example, exosomal miR-497 effectively inhibits the expression of VEGF-A and suppresses tumour growth. Therefore, it may be a tool for the development of lung cancer therapies [80].

## Microenvironmental regulation by exosomes

The premetastatic TME (pre-metastatic niche) in lung cancer is an important cause of induction of metastasis. Primary lung cancer-derived exosomal RNAs promote neutrophil recruitment by activating TLR3 in lung epithelial cells via the NF-kB and MAPK pathways [81]. TGF-β has long been a pivotal adaptor in the lung cancer TME. It contributes to the development of cancer and directly impairs T cell immune function [82, 83]. Recent studies have found that TGF-β also has a regulatory effect on exosomal factors. TGF-β-pretreated A549 cell-derived exosomes increase the expression of MMP2 at the gene and protein levels and regulates vascular permeability [84]. Thyroid transcription factor-1 (TTF-1) is mainly expressed in lung adenocarcinomas and regulates angiogenesis activity in the TME. Both vascular endothelial growth factor (VEGF) and granulocyte–macrophage colony-stimulating factor (GM-CSF) are regulated by TTF-1, which reprograms lung adenocarcinomas that secrete TME factors in angiogenesis [85]. Leucine-rich-alpha2-glycoprotein 1 (LRG1) is upregulated in NSCLC tissues and promotes NSCLC cell invasion. NSCLC cell-derived exosomes with LRG1 activate the TGF-β signalling pathway and thus promote angiogenesis [86]. CAF-derived exosomes also play a role in the microenvironment of lung cancer by promoting EMT. MiR-210 secreted by CAF-exosomes could target UPF1, promote the PTEN/PI3K/AKT signalling pathway, and contribute to NSCLC invasion by regulating EMT factors, such as E-cadherin, N-cadherin, and vimentin [87]. These studies suggest that exosomal RNAs and proteins are novel therapeutic targets and predictive markers of tumour metastasis in lung cancer.

**Fig. 1:**
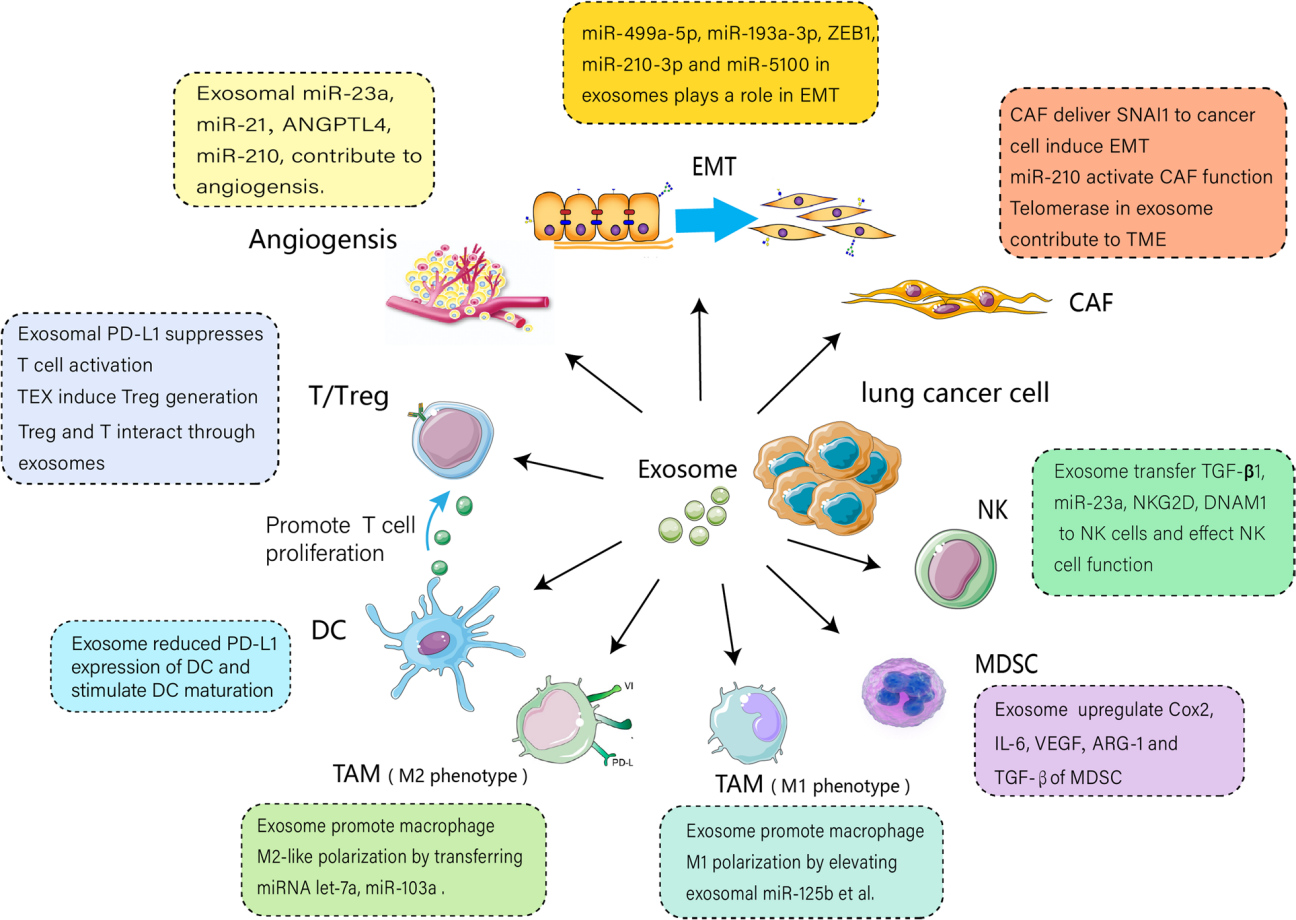
The effect of exosome from lung cancer cell on TME

Table 1 shows the interaction of exosomes between different cells in TME of lung cancer and the effect of exsome from lung cancer cell on TME was illustrated on Fig. 1.

**Table 1:** The interaction of exosomes between different cells in TME of lung cancer

Parental cells	Exosomal cargos	Exosome/Cargos formation mechanism	Recipient cells	Target	Biological/clinical relevance	References
macrophages	let-7a-5p	/	A549	BCL2L1	Induced A549 lung cancer cell death and altered expression of MYC, EGFR, and Vimentin	[14]
A549	let-7a	Hypoxia	Macrophages	Insulin-Akt-mTOR pathway	Enhanced macrophage recruitment, promoted M2-like polarization	[31]
CL1-5	miR-103a	Hypoxia	Macrophages	PTEN and Akt/Stat3	Increased M2-type polarization, enhanced cancer progression and tumor angiogenesis	[33]
SK-LU-1	miR-125b et al	Double-targeted (wild-type p53 and microRNA-125b) transfection	J774 macrophages	/	J774 macrophages repolarized towards M1 phenotype	[34]
A549/LLC	/	/	DCs	PD-L1	Reduced the expression of PD-L1 of DCs, down-regulated the population of Tregs	[37]
A549	/	Rab27a	DCs, CD4^+^ T-cell	/	Induced higher levels of cytokines (IL-1β, TNF-α, RANTES), promoted CD4^+^ T-cell proliferation	[40]
IGR-Heu	TGF-β, miR-23a	Hypoxia	NK	NKG2D	Decreased the cell surface expression of the activating receptor NKG2D, thereby inhibiting NK cell function	[44]
LLC	miR-21、miR-29a	/	TAM	TLR8	Activated NF-κB and promoted the secretion of inflammatory cytokines TNF-α and IL-6	[27]
NK	DNAM1	IL-2/IL-15	Lung tumor cell	DNAM1-ligands	Involved in NK mediated cytotoxicity and result in killing of tumor cells	[47]
LLC	PD-L1	/	DC	/	Blocked the differentiation of DCs and increased the rates of Treg	[49]
Treg	let-7d	Rab27a and Rab27b	Th1	Cox-2	Suppressed Th1 cell proliferation and cytokine secretion	[53]
Lung cancer cell	EGFR	/	DCs	/	Induced tolerogenic DCs, which effected on Th0 to produce Treg	[54]
KRAS mutant NSCLC	KRAS protein	K-ras gene mutant	CD4^+^ T-cell	IFN signaling	Induced CD4^+^ T phenotypic conversion to FOXP3^+^ Treg-like cells that are immune-suppressive	[56]
CAFs	SNAL1	/	Lung tumor cell	E-ca, a-SMA, vimentin	Induced metastasis and drug resistance in NSCLC	[13]
Mesenchymal lung cancer cell	ZEB1 mRNA	/	Bronchial epithelial cell	/	Transferred chemoresistance and mesenchymal phenotypes to bronchial epithelial cell via ZEB1 mRNA	[63]
BMSCs	miR-193a-3p, miR-210-3p and miR-5100	Hypoxia	Epithelial and mesenchymal cell	STAT3 signal	Promoted cancer cell invasion and EMT	[15]
A549/SPC-A-1-BM	miR-499a-5p	/	Epithelial and mesenchymal cell	mTOR signal	Enhanced cell proliferation, migration and EMT via mTOR pathway	[64]
CL1-5 cells	miR-23a	Hypoxia	Endothelial cells	PHD 1 and 2	Promoted angiogenesis and tumour growth	[76]
CL1-5 cells	miR-23a	Hypoxia	Endothelial cells	ZO-1	Increased vascular permeability and cancer trans-endothelial migration	[76]
A549	ANGPTL4	γ-ray irradiated or hypoxic conditions	Human umbilical vein endothelial cells	/	Contributed to the migration of NSCLC as well as the angiogenesis of HUVECs	[77]
Lung cancer cell	miR-210	/	CAFs	TET2 and JAK2/STAT3	Promoted release of proangiogenic factors VEGF, MMP9 and FGF2	[68]
Cigarette smoke extract (CSE)-transformed human bronchial epithelial (HBE) cells	miR-21	STAT3	(Human bronchial epithelial)HBE, human umbilical vein endothelial cells (HUVEC)	STAT3	Lead to STAT3 activation, which increases VEGF levels in recipient cells	[79]
hek293t	miR-497	/	A549, HUVECs	VEGF-A, HDGF, CCNE1	Contributed to tumor growth and angiogenesis	[80]
LLC2	miR-126-3p, miR-27b, miR-320, and miR-342-3p	/	/	MDSCs	Activated their immunosuppressive functions	[60]
Lung cancer cell	miR-9	/	HUVECs	SOCS5/JAK-STAT pathway	Regulated SOCS5-JAK-STAT pathway and promoted endothelial cell migration and tumour angiogenesis	[65]
A549	miR-210	TIMP-1	HUVECs	/	Downstreamed targets of miR-210: FGFRL1, E2F3, VMP-1, RAD52 and SDHD	[78]
CAFs	miR-210	/	NSCLC	UPF1	Promoted EMT by activating PTEN/PI3K/AKT pathway	[87]
H1437 and H2073	miR-142-3p	/	Endothelial and fibroblast cells	TGFβ signaling	Promoted angiogenesis through inhibition of TGF-βR1	[66]
A549	EGFR	/	Endothelial cells	MAPK and Akt pathways	Induced and modulated tumor angiogenesis	[120]
PC14HM	/	/	HBECs	/	Induced vimentin expression and EMT in HBECs	[121]
Human brain microvascular endothelial cells	S100A16 protein	/	SCLC	Δψm, prohibitin (PHB)-1	Facilitated the survival of SCLC cells through modulating the mitochondrial function	[16]
A549	lnc‐MMP2‐2	TGF‐β	Vascular endothelial cell	/	Increased vascular permeability	[84]
LLC	Exosomal RNAs	/	Lung epithelial cells	TLR3	Provided potential targets to control cancer metastasis	[81]
LLC	LRG1	A549	NSCLC, vein endothelial cells	TGF-β signaling	Promoted angiogenesis	[86]
B cell	CD39 and CD73	Hypoxia-inducible factor-1α	CD8^+^ T cell	/	Impaired CD8^+^ T cell responses	[20]
H1792 and HCC44	VEGF	TTF-1	endothelial cell	GM-CSF/VEGF axis	Contributed to angiogenesis	[85]

## Roles of exosomes in treatment resistance

The progression of multidrug resistance is the major obstacle to maintain effectiveness of chemotherapy in lung cancer [88]. The TME is enriched with DDP-resistant lung cancer cell-derived exosomes. These exosomes with miR-100-5p could be absorbed by other lung cancer cells and exerted their DDP resistance function. Qin and colleagues explained that cancer cells could be regulated by miRNAs themselves and the TME in vitro [89]. These results have been verified in vivo using a gemcitabine-resistant (GR) cell line. GR cell-derived exosomes transfer miR-222-3p, thereby contributing to malignancy in NSCLC by targeting SOCS3 after endocytosis. Additionally, the miR-222-3p level in serum exosomes may predict gemcitabine sensitivity and worse prognosis in NSCLC [90]. Radiotherapy is another promising method to completely cure localized non-metastatic cancer, in addition to surgical resection. The level of serum exosomal miR-208a could be elevated by X-ray irradiation in patients with NSCLC. miR-208a could target p21 and activate the AKT/mTOR pathway, thereby promoting cell proliferation and inducing radio-resistance. Therefore, exosomal miR-208a could serve as a target to enhance the efficacy of radiotherapy [91]. Some miRNAs have a radiosensitising effect. For example, miR200c and miR148b both inhibit proliferation by regulating EMT and possess radiosensitising effects [92, 93]. Wu et al. found that exosomal miR-96 isolated from H1299 enhanced cell malignancy and cisplatin resistance by targeting LIM-domain only protein 7 (LMO7) [94]. The role of exosomes in resistance to targeted therapy has also been investigated. Exosomal PLAUR regulates the EGFR/AKT/survivin signalling pathway and induces gefitinib resistance [95]. Similarly, exosome-derived miR-564 and miR-658 also induce gefitinib resistance, and targeting these miRNAs may reverse resistance [96]. Additionally, UCA1 in exosomes may be another therapeutic target for patients with EGFR-positive lung cancer. Resistance to tyrosine kinase inhibitors has become a main factor limiting clinical efficacy for patients with advanced-stage NSCLC. Zhang et al. confirmed that serum lncRNA RP11‑838N2.4 expression was improved in patients with resistance to erlotinib. Furthermore, the knockdown of the lncRNA RP11‑838N2.4 could promote erlotinib cytotoxicity [97]. Another target for the reversal of erlotinib resistance is the lncRNA H19. H19 from exosomes can regulate ATG7 expression by binding to miR-615-3p, thereby promoting erlotinib treatment resistance. Therefore, H19 may be another target for patients with erlotinib resistance [98].

## Exosomes as diagnostic and therapeutic targets

Exosomes in the TME may be novel diagnostic and therapeutic targets for NSCLC. YKT6 in lung cancer cells regulates exosome release. A clinical study has shown that in NSCLC, YKT6 in tumour samples is associated with a shorter disease-free survival and overall survival [12].

Various lncRNAs have been identified as promising therapeutic targets. MiR-96 is a candidate serum biomarker and therapeutic target for NSCLC. Melanoma differentiation-associated gene-9 (MDA-9)/Syntenin is another therapeutic target for lung adenocarcinoma; this locus promotes cancer invasion and metastasis as a key regulator of Slug and Slug-mediated EMT [99]. NSCLC cell-released exosomal miR-619-5p could promote angiogenesis by targeting RCAN1.4 and induced the growth and metastasis of lung cancer cells. A clinical study found that miR-619-5p was expressed at higher level in exosomes isolated from the plasma of patients with NSCLC compared with the level in healthy individuals[100]. Exosome-derived EGFR has also recently been identified as a diagnostic marker for NSCLC based on its high expression in plasma exosomes of patients with NSCLC, and it can be specifically captured by CD81 antibodies [101]. In terms of angiogenesis, radiation-induced ANGPTL4 derived from exosomes contributes to angiogenesis and lung cancer cell migration, suggesting that exosomal ANGPTL4 is a therapeutic target [77].

## Exosomes of the TME as prognostic biomarkers for lung cancer

Exosomes are promising tools for tumour diagnosis and treatment [4, 8]. MicroRNAs in exosomes may be diagnostic biomarkers for lung cancer. Zhang et al. found that the downregulation of exosomal let-7a-5p with the upregulation of the target gene *BCL2L1* could be a useful biomarker for poor survival in patients with lung adenocarcinoma [102]. A large number of studies have shown that miR-21 is associated with survival and is actively involved in the modulation of malignant transformation and progression in NSCLC [103]. Exosomal miR-106b may elevate the expression of MMP-2 and MMP-9, which play a role in angiogenesis in the TME, and may enhance the invasive ability of NSCLC. Additionally, serum exosomal miR-106b in patients with lung cancer is associated with the TNM stage and lymph node metastasis [104]. Another TME factor with prognostic value is NEK2, a target of miR-486-5p, which is associated with the TNM stage. Exosomal miR-486-5p is downregulated in the serum of patients with lung cancer and contributes to tumour formation via effects on the EMT [105]. High levels of MDA-9/Syntenin and Slug are related to poor overall survival in patients with lung adenocarcinomas [99]. Exosomes are also a new tool for gene sequencing of targeted therapies. Clinical studies have shown that RNA/DNA detection in exosomes can improve the detection rate of patients who are positive for EGFR mutations (including the L858R and T790M mutations) [106, 107].

Table 2 expresses a summary of exosomes related experiments and studies contributing to radiotherapy, chemotherapy, targeted therapy, diagnosis and prognosis.

**Table 2:** Exosomes related experiments and studies contributing to radiotherapy, chemotherapy, targeted therapy, diagnosis and prognosis

Exosomal cargos	Clinical significance	Parental cells	Targets of action	Biological/clinical relevance	References
RP11-838N2.4	Upregulated in patients with erlotinib resistance	HCC827, HCC4006	FOXO1	Knockdown of lncRNA RP11-838N2.4 promoted erlotinib-induced cytotoxicity	[96]
let-7a-5p/BCL2L1	Predictive biomarkers for poor survival	Lung adenocarcinoma	BCL2L1	Downregulation of let-7a-5p and elevation of BCL2L1 as predictive biomarkers for poor survival	[14]
miR-486-5p	Present difference in different TNM stages	Lung adenocarcinoma	NEK2	MiR-486-5p was responsible for cell cycle arrest as well as the inhibition of cell proliferation and metastasis via targeting NEK2	[105]
MDA-9/Syntenin	Associated with poor overall survival	H1299, CL1–5, CL141	Slug	MDA-9/Syntenin acted as a pivotal adaptor of Slug and it transcriptionally enhanced Slug-mediated EMT	[99]
miR-619-5p	Diagnostic indicator	A549, H460	RCAN1.4	MiR-619-5p targeted RCAN1.4 and promoted angiogenesis. Exosomal miR-619-5p can serve as a diagnostic indicator	[100]
TTF-1/ NKX2-1	Favorable prognosis	H1792, HCC44	GM-CSF/VEGF	Reprogrammed lung adenocarcinoma secreted TME factors in angiogenesis	[85]
EGFR	Diagnostic biomarker	Lung cancer cell	CD81	EGFR expression using a targeted ELISA for lung cancer diagnosis	[101]
miR-106b	Diagnostic biomarker and drug target	Lung cancer cell	PTEN	MiR-106b targeted PTEN, promoted cancer cell migration and invasion	[104]
LRG1	Potential therapeutic target	A549	TGF-β	LRG1 promoted angiogenesis via TGF-β signaling	[86]
Human brain microvascular endothelial cells	Prognosis of brain metastases	/	(PHB)-1 in Δψm	S100A16 facilitated the survival of SCLC cells through modulating the mitochondrial function	[16]
ANGPTL4	Diagnostic biomarker and therapeutic target	A549	Angiogenesis	ANGPTL4 contributed to the migration of A549 cells as well as the angiogenesis of HUVECs	[77]
miR-100-5p	New insights of DDP resistance	A549	mTOR	miR-100-5p was absorbed by lung cancer cells and displayed DDP resistance function	[89]
miR-222-3p	Pognostic biomarker for predicting gemcitabine sensitivity	A549	SOCS3	Sera miR-222-3p as a potential prognostic biomarker for predicting worse prognosis and gemcitabine sensitivity	[90]
miR-208a	Affect the radio-sensitivity of NSCLC	Lung cancer cell	p21	MiR-208a induced radio-resistance via targeting p21 and AKT/mTOR pathway	[91]
MicroRNA-200c	Increased the radio-sensitivity	Lung cancer cell	EGFR	MiR-200c improved efficacy of radiotherapy via controlling cancer pro-survival signaling and EMT	[92]
MicroRNA-148b	Enhance the effects of radiotherapy	A549	ROCK1	MiR-148b inhibited NSCLC cell proliferation and the EMT, and increased the radio-sensitivity by inhibiting ROCK1	[93]
miR-96	Serum biomarker of malignant lung cancer	Lung cancer cell/H1299	LMO7	MiR-96 promoted lung cancer progression by targeting LMO7	[94]
PLAUR	Therapeutic target for gefitinib-resistant	Gefitinib-resistant PC9R cells	EGFR/p-AKT/survivin pathway	PLAUR induced geftinib-resistance through EGFR/p-AKT/survivin signaling pathway	[95]
miR-564 and miR-658	Therapeutic target of resistance against gefitinib	Gefitinib-resistant PC-9/ZD	PC-9 cells	MiR-564 and miR-658 induced drug resistance in sensitive cells	[96]
lncRNA H19	Decreased the erlotinib resistance	HCC827/A549	miR-615-3p/ATG7 axis	H19 facilitated erlotinib resistance in NSCLC via miR-615-3p/ATG7 axis	[98]
Circulating exosome	Higher sensitivity and specificity for T790M detection	NSCLC of patients	/	The combination of exoRNA/DNA and cfDNA for T790M detection has higher sensitivity and specificity	[107]
YKT6	Impact prognosis of resected NSCLC patients	A549	miR-134 and miR-135b	YKT6 regulated exosome release and is in turn regulated by miR-134 and miR-135b	[12]
Circulating exosome	As a prognostic factor for NSCLC and correlates with tumor stage	NSCLC of patients	/	Plasma exosome was associated with tumour stage and poorer overall survival	[122]

## Exosomes as biomarkers for liquid biopsy of lung cancer

Liquid biopsy is a diagnostic procedure that describes information about cancer-derived substances obtained from blood sample. The sample information of the liquid biopsy mainly comes from: circulating tumour cells (CTCs) of blood sample, circulating cell-free DNA (cfDNA) released into the blood from tumour cells and normal cells and cell-free RNA (cfRNA) enriched in exosomes from tumour cells [108]. It makes up for the inadequacy of tissue biopsy in advanced NSCLC patients with poor state, lung cancer very early detection, the side effects of interventional biopsy procedures, insufficient tissue quantity, and false positive molecular detection analysis [109]. Liquid biopsy is expected to be another important means for lung cancer diagnosis and gene sequencing analysis. Clinical diagnosis of patients with lung cancer pneumomeningeal metastasis (LM) is difficult. Serum exosomal miR-483-5p and miR-342-5p may play a role in the diagnosis of these patients and may replace cerebrospinal fluid in predicting LM of NSCLC [110]. Similarly, exosomal miR-17-5p expression is significantly up-regulated in NSCLC patients. Based on this, the combination of exosomal miR-17-5p, carcinoembryonic antigen (CEA), cytokeratin 19 fragment (CYFRA21-1) and squamous cell carcinoma antigen (SCCA) is considered to be a newly developed diagnostic panel of NSCLC [111]. Extracellular Vesicle (EVs)-Derived CD5L protein expression was detected to be associated with cancer tissue in clinic. This result suggests that CD5L may be another potential biomarker for noninvasive diagnosis of NSCLC. The EVs were detected to be about 76-194 nm in diameter, which included the category of exosomes [112]. In addition, the combination of miR-21-5p, miR-223-3p, miR-155-5p and miR-126-3p may be a potential diagnostic biomarker for lung cancer [113]. In terms of EGFR mutation detection, combining exosomal RNA and cfDNA (exoNA) can improve the sensitivity of EGFR mutation detection in liquid biopsy from NSCLC patients. The sensitivity of EGFR mutation positive detection by exoNA method can reach 98% [114]. Exosomal nucleic acids are more sensitive to the identification of associated mutations than cfDNA. This is of great significance for the selection of targeted therapy [115].

PD-L1 in serum exosomes can be used as a quantitative factor for tumour PD-L1 status, which may be helpful in predicting the clinical outcome of anti-PD-1 therapy in NSCLC patients [116]. With the development of immunotherapy, microsatellite instability (MSI) and mismatch repair deficiency (MMRD) tumours have high immunogenicity and have been used as predictive biomarkers for PD-1 inhibitor efficacy and demonstrated in clinical trials of anti-PD-1 therapy [117]. MSI can also be diagnosed by analysis of miRNA-mRNA network in exosomes biomarker sources and therapeutic applications [118]. Detection of MSI and MMRD gene sequences in exosomes by liquid biopsy is of great significance for PD-1 and PD-L1 immunotherapy [119].

## Conclusion

Functional studies of non-coding RNAs and proteins in exosomes provide new insights into reshaping the TME. In terms of clinical application, a large number of non-coding RNAs and proteins have been found in exosomes, which are expected to become an indispensable tool for the diagnosis and prediction of lung cancer in clinic. However, there remains a lack of clinical studies with large samples to provide evidence support. It is particularly important to identify the precise components that act key roles in tumour processes. Ongoing experimental and clinical studies of exosomes may provide new ideas for improvement of TME and treatment of lung cancer.

## Data Availability

The primary data for this study is available from the authors on direct request.

## Abbreviations

CAF: Cancer-associated fibroblast
DC: Dendritic cell
ECM: Extracellular matrix
EMT: Epithelial–mesenchymal transition
NK cell: Natural killer cell
NSCLC: Non-small cell lung cancer
SCLC: Small cell lung cancer
TAM: Tumour-associated macrophage
TME: Tumour microenvironment
Treg: Regulatory T cells
